# Smartphone Use—Influence on Posture and Gait during Standing and Walking

**DOI:** 10.3390/healthcare11182543

**Published:** 2023-09-14

**Authors:** Marius Brühl, Jamil Hmida, Fabian Tomschi, Davide Cucchi, Dieter C. Wirtz, Andreas C. Strauss, Thomas Hilberg

**Affiliations:** 1 Department of Sports Medicine, University of Wuppertal, Moritzstraße 14, 42117 Wuppertal, Germany; marius.bruehl@uni-wuppertal.de (M.B.); hilberg@uni-wuppertal.de (T.H.); 2 Department of Orthopaedics and Trauma Surgery, University of Bonn, Venusberg Campus 1, 53127 Bonn, Germany

**Keywords:** spine, surface topography, motor control, biomechanics, gait analyses, mobile, treadmill

## Abstract

Prolonged gaze at a smartphone is characterized by pronounced flexion of the cervical spine and is associated with health risks. In addition, it is suspected that smartphone distraction could lead to gait changes. Therefore, the aim of this study was to detect smartphone-associated postural changes at thoracic and lumbar levels as well as gait changes. Spinal analysis was performed prospectively in 21 healthy men using the DIERS 4Dmotion^®^Lab in a controlled crossover design to evaluate posture-associated parameters while standing and walking. The examination sequence provided three randomized gaze directions: GN = Gaze Neutral; S1H = Smartphone one-handed; S2H = Smartphone two-handed. Results reveal a higher vertebra prominens (VP)-flexion in S1H (23.8° ± 6.9°; *p* ≤ 0.001) and S2H (22.4° ± 4.7°; *p* ≤ 0.001) compared to GN (17.6° ± 3.8°). Kyphosis angles were also different with higher values observed in S1H (58.8° ± 5.8°; *p* ≤ 0.001) and S2H (61.6° ± 4.9°; *p* ≤ 0.001) compared to GN (49.1° ± 4.6°). During walking, similar results were observed in kyphosis angles. No differences were observed in gait during smartphone use (*p* = 0.180–0.883). The study revealed a significantly increased inclination of the lower cervical and thoracic spine during smartphone use. However, the inclination was larger during S2H. Standing or walking conditions did not affect the measurement outcomes. Long-term smartphone use associated with a larger inclination of the cervical and thoracic spine might result in increased pressure and shear forces acting on vertebral bodies, intervertebral discs, and muscles, which potentially increases the risk of spinal pain and disease.

## 1. Introduction

There are currently 3.6 billion smartphone users worldwide. According to forecasts, the number is projected to increase to as much as 4.5 billion by 2024, indicating that more than half of the global population owns a smartphone [1]. In Germany, the proportion of smartphone users was as high as 88.8% in 2021 [2]. In particular, more than 90% of the 14–59 age group used a smartphone [2]. The average time daily spent using a smartphone is about 3 h [3]. Predominantly, the smartphone is used for media consumption as well as communication [4].

The effects of this exposure are multifaceted and, in some cases, not well investigated. E.g., adolescents who exhibited increased smartphone use, specifically social media, showed an increased risk for depressive disorders [5]. Biomechanically, prolonged smartphone gaze is characterized by pronounced cervical spine flexion of up to 45° [6]. The head’s own mass of 4.5 to 5.4 kg increases when the cervical spine is in flexion so that the acting torque generates a load of up to 22.2 kg. [7]. In addition, mobile device use is related to a 1-week prevalence rate of 17.3 to 67.8% for musculoskeletal complaints in the neck [8]. This could be due to the reported flexion of the cervical spine [9], which is associated with increased muscle activity of the splenius muscle, erector spinae muscle, and trapezius muscle. Surface electromyography measurements revealed 189% to 295% higher muscle activity under standing conditions. A further increase of 21.2% to 41.7% was measured under walking conditions while using the smartphone compared to normalized amplitudes of upright standing without using a smartphone [10].

Considering the changed posture in the cervical spine, it is reasonable to assume that structures of the spine lying further caudally also exhibit a changed posture. This was demonstrated under standing and walking conditions and a significantly increased kyphosis angle of the thoracic spine and lordosis angle of the lumbar spine while writing a text on the smartphone was observed [11]. It was also reported that the lumbar lordosis angle parameter showed larger deviations when walking with a smartphone held by two hands, in comparison to using just one hand. However, apart from this observation, no differences were measurable in the context of comparing unilateral versus bilateral smartphone use [11].

That the use of a smartphone not only changes the posture but also the gait is supported by a significant change in speed, stride length, foot rotation, and step width [12,13]. These changes in gait might be due to increased uncertainty and attempts to reduce the risk of stumbling or even falling associated with the need to divide attention between the simultaneous cognitive and motor tasks inherent in mobile phone use while walking [13].

Yet, there is a lack of knowledge regarding the alterations occurring in the thoracic and lumbar spine during smartphone usage within controlled and standardized conditions, and the potential implications this could pose for health. Therefore, the purpose of this study was to detect smartphone-use-associated postural changes in the spine under standing and walking conditions while reading a text on a one- or two-handed held smartphone and to demonstrate changes in gait to answer the following questions:

(1) Does looking at a smartphone lead to a change in the sagittal spine parameters VP-flexion, kyphosis, and lordosis angles in the thoracic and lumbar spine during standing and walking?
(2) Does reading a text on a smartphone held one-handed or two-handed result in different postures?
(3) Does reading a text on a smartphone result in altered gait patterns?

Based on the above-mentioned considerations, it is hypothesized (1) that reading a text on a smartphone leads to alternations in cervical, thoracic, and lumbar spine posture; (2) that due to adjustments in gaze direction resulting from holding the smartphone with two hands, there will be larger flexion angles compared to holding it with one hand; (3) that a change in gait pattern occurs, resulting in adjustments such as shorter steps, increased step width, and higher cadence.

## 2. Materials and Methods

### 2.1. General Study Design

**Figure 1:**
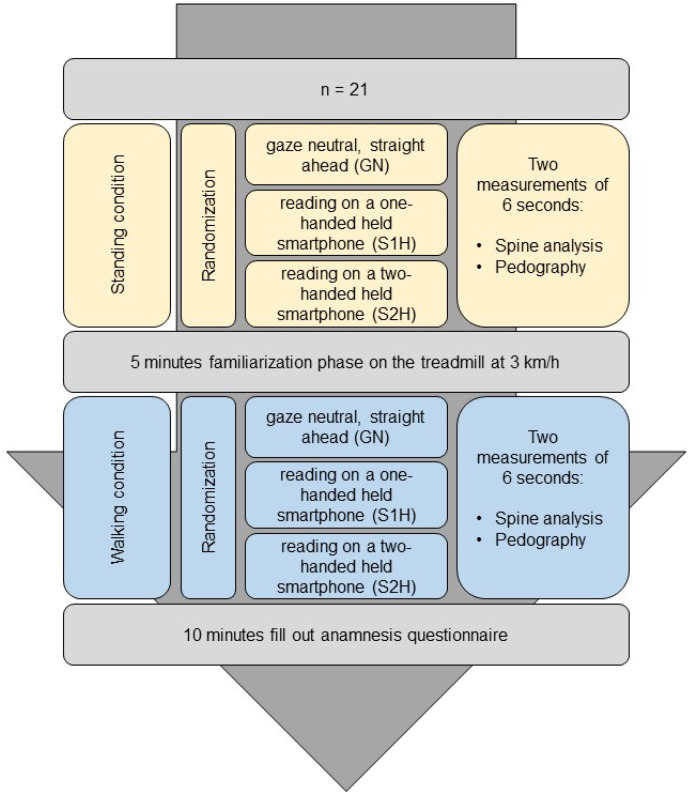
Study design of the investigation. GN = Gaze neutral; S1H = Smartphone held one-handed; S2H = Smartphone held two-handed.

This crossover study was based on a quantitative cross-sectional design examining differences in spinal kinematics and gait during gaze manipulation (Figure 1). The independent variables consisted of three conditions: (1) gaze neutral, i.e., straight ahead (GN), (2) reading on a smartphone held in one hand (S1H), and (3) reading on a smartphone held in two hands (S2H). These conditions were performed under standing as well as walking conditions. The dependent variables describe the spinal kinematic and gait analysis parameters of the measurement instrument, which are described below.

### 2.2. Participants

A total of 21 healthy male subjects were recruited, who used a smartphone daily (age (years): 25.1 ± 2.2 (21–31) (mean ± SD (min.–max.)). The anthropometric data of the subjects were: height (cm) (181.6 ± 7.5 (170–198)), body mass (kg) (81.2 ± 10 (56–105)), body mass index (24.7 ± 3.3 (18.7–33.9)), handedness (self-disclosure) (20 right-handed 1 left-handed), and smartphone-handedness (17 right-handed 4 left-handed). Included were male subjects between 18 and 40 years without acute back pain or musculoskeletal injury in the past 3 months. Individuals with serious internal or orthopedic diseases such as Bechterew’s disease, Scheuermann’s disease, or rheumatoid arthritis were excluded. Furthermore, the subjects must not have had sore muscles or have exercised for 24 h prior to the study. All participants gave their written informed consent according to the declaration of Helsinki, and the study protocol was approved by the local ethics committee.

### 2.3. Data Collection

Each subject completed an anamnesis questionnaire (German pain questionnaire [14]) prior to the examination to screen for musculoskeletal disorders and pain. For the measurement, the subjects were asked to undress down to their underpants, and reflective markers were applied to predefined areas of the body. Subsequently, standing measurements were conducted, for which the subject stood on the treadmill and looked straight ahead at the wall 1.5 m away without a marker. The subject was instructed to walk a few steps on the spot and then stand still to assume the most natural posture. The measurement condition GN was considered as a control measurement without any manipulation of the gaze direction. During the measurement conditions S1H and S2H, a standardized 3.5-inch smartphone (iPhone 4, Apple Inc., Cupertino, CA, USA) was used, which displayed the text of the fairy tale “Alice in Wonderland” [15]. The measurement condition S1H was based on holding the smartphone with only the habitual hand. In the S2H measurement condition, the subject was supposed to hold the smartphone two-handed in front of the body, without any further instructions. These six measurement conditions were randomly assigned to subjects using a randomization software (http://www.randomization.com/ (accessed on 8 February 2021)). Then, the walking measurements were conducted starting with a 5 min familiarization phase on the treadmill at 3 km/h and no incline [16]. Afterwards, the already known measurement conditions were repeated while walking at 3 km/h. Before the start of each condition, there was a familiarization phase of 2 min and after each condition, there was a 2 min break during which the participant exited the treadmill [17].

### 2.4. Data Acquisition and Analyses

**Figure 2:**
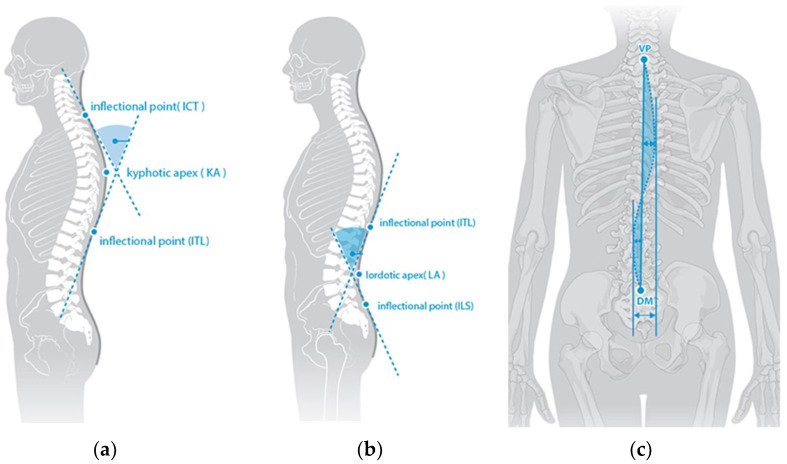
(**a**) Explanation of the (**a**) thoracic kyphosis angle ICT-ITL (max) (**b**) lumbar lordosis angle ITL–ILS (max) (**c**) lateral deviation VP-DM (RMS) [mm]. (DIERS international GmbH, 2022). VP = vertebra prominens; DM = dimple middle; ICT = cervico-thoracic junction; ITL = thoraco-lumbar junction; ILS = lumbo-sacral junction. The illustrations are reproduced with permission from DIERS international GmbH.

The measurement system used in this investigation to evaluate spine and gait characteristics was the DIERS 4Dmotion^®^Lab surface topography system (DIERS international GmbH, Schlangenbad, Germany). The system consists of the components DIERS formetric for the measurement of the spine and DIERS pedogait for the pedobarography. A comprehensive analysis of the participants’ posture under standing as well as walking conditions was performed. The spine measurement is based on the mathematical method of triangulation. Using 10 mm reflective markers on specific anatomic landmarks (vertebra prominens and spina iliaca posterior superior right and left), the system generates a topographic image of the subject’s back and constructs the underlying spine using the software DICAM3 v. 3.11 (DIERS international GmbH, Schlangenbad, Germany). The primary parameters evaluated in this study for spine measurement were the thoracic kyphosis angle, lumbar lordosis angle, and flexion of the vertebra prominens in the sagittal plane as well as the lateral deviation in the frontal plane. These parameters were calculated by the software using different reference points, which is described as reliable [18]. The kyphosis angle is calculated from the angle between the surface tangent at the cervico-thoracic junction (ICT) and thoraco-lumbar junction (ITL) inflection points (Figure 2a). The lordosis angle refers to the inflection points thoraco-lumbar junction (ITL) and lumbo-sacral junction (ILS) (Figure 2b). The lateral deviation is calculated as the mean square deviation of the midline of the spine from the direct connection between the vertebra prominens (VP) and the center of the lumbar dimples (DM) (Figure 2c). Under standing conditions, the measurement lasted 6 s at 2 frames per second, and under walking conditions, the measurement lasted 5 s at 60 frames per second. The gait analysis took place simultaneously with the spine measurement on a Zebris FDM-T treadmill (Zebris Medical GmbH, Isny, Germany). Using the capacitive pressure measuring plate (size: 1084 × 474 mm) integrated into the treadmill with a resolution of 1.4 sensors per 1 cm^2^ and a recording frequency of 120 Hz, the pressure distribution of the feet is displayed. The parameters of the gait analysis evaluated in this study were stride length, cadence, step width, and foot rotation, which were calculated on the basis of pedobarography and the resulting compressive forces.

### 2.5. Statistics

Statistical analysis was performed using the IBM© SPSS 29 software (Armonk, NY, USA) statistical program for Windows. All data were tested for normal distribution using the Kolmogorov–Smirnov test and for variance homogeneity using Levene’s test with no need for further transformation. To compare the different gaze directions under standing and walking conditions, inductive statistics were used to perform repeated-measures ANOVA. Sphericity was checked by the Mauchly test, and the Greenhouse–Geisser correction was applied when necessary. Effect sizes are presented as partial eta-squared (η^2^) with values of 0.01 representing small, 0.06 a medium, and ≥0.14 a large effect, respectively [19]. If a significant effect was observed resulting from the ANOVA, Bonferroni post hoc analyses were conducted to detect significant differences between separate conditions. In addition, post hoc power (1 − β) was calculated using G*Power (version 3.1.9.4, Heinrich Heine University, Düsseldorf, Germany). The level of significance was set at *p* < 0.05 (95% confidence interval) for all statistical analyses.

## 3. Results

### 3.1. Results of the Spine Analysis

In this study, 21 healthy males were included. Due to technical problems with the spine analysis, the data of the spine measurements are missing for one subject. Results of comparisons of kyphosis angle, lordosis angle, VP-flexion, and lateral deviation under standing and walking conditions for GN, S1H, and S2H are presented in Table 1.

### 3.2. Results of the Gait Analysis

The gait analysis did not show any significant differences between gaze directions GN, S1H, and S2H for any of the mentioned parameters of stride length, cadence, step width, and foot rotation (*p* = 0.180–0.883) (Table 2).

## 4. Discussion

The aims of this study were threefold, and the main results reveal that (1) specific cervical, thoracic, and lumbar spine parameters (VP-flexion, kyphosis, and lordosis angles) are altered during smartphone use compared to the control condition. This was observed during standing and walking; (2) using a smartphone one-handed leads to changes in kyphosis angle, VP-flexion, and lateral deviation compared to two-handed use; and (3) reading a text on a hand-held smartphone while walking slowly does not lead to any changes in gait characteristics.

The increased inclination of the lower cervical and thoracic spine was particularly observed during the two-handed use, where this effect was even more expressed compared to the one-handed use. Significantly increased VP-flexion and kyphosis angles were shown compared to the control condition GN. This has already been shown for the parameter VP-flexion, and the present results are in line with current studies [6,7].

In contrast, thoracic spine flexion during smartphone use has been investigated rarely. The results of the study presented herein reveal that the control condition GN showed a kyphosis angle of 49.1° ± 4.6° under standing and 47.6° ± 4.6° under walking conditions. These values correspond to the norm values of symptomatic-free adults [20]. When subjects read a text on the smartphone, the kyphosis angle increased under standing S1H conditions to 58.8° ± 5.8° and under walking S1H conditions to 56.5° ± 5.5°. Under S2H standing conditions, angles increased to 61.6° ± 4.9° and under S2H walking conditions to 58.5° ± 5.6°. This amplification of the kyphosis angle suggests that the threshold value of a round back of 50° was exceeded, especially during two-handed use [21]. These results demonstrate that the changed gaze direction does not result exclusively from increased flexion of the cervical spine but is also produced by the thoracic spine following the spine caudally. This posture results in increased pressure and shear forces acting on the vertebral bodies, intervertebral discs, and muscles, thus increasing the risk of spinal pain and disease [22]. Looking at the lumbar spine, a not statistically significant increased lumbar lordosis angle of plus 2.5° was measured during S1H and 2.1° during S2H under standing conditions compared to GN. It was previously shown that the lumbar lordosis angle during phone calls and two-handed texting on a smartphone increased [11]. A normative lumbar lordosis angle during standing is between 32° and 37° [20,23]. During GN, the lordosis angle was within this range (35.5° ± 7.1°). S1H as well as S2H exceed this norm by 1° and 0.6° with 38° ± 7.6° and 37.6° ± 11.2°, respectively. However, these statistically non-significant differences might suggest that lumbar spine posture can be affected by reading on a smartphone. A lumbar spine angle increment of 2° results in an increased pressure load on the intervertebral discs and may be associated with an increased risk of musculoskeletal complaints if this posture is adopted repeatedly [24,25].

In addition to the changes on the sagittal plane, the spinal parameter lateral deviation on the frontal plane stood out. The lateral deviation is considered normal up to 5 mm [21] and results of the present study reveal that lateral deviation is increased during S1H in standing and walking conditions. Similar findings have already been presented in another study in which the smartphone was held to the ear to make a phone call [11]. Therefore, these outcomes imply that the manner in which the smartphone is held also impacts the posture of the thoracic spine on the frontal plane. One explanation for the increased lateral deviation during S1H could relate to the arm posture and the accompanying acting forces of the muscles. While holding the smartphone one-handed, the arm describes an anteversion and rotation with simultaneous flexion and supination of the elbow by approximately 90°. This is accompanied by a protraction of the shoulder, which in turn causes an anterolateral movement of the scapula. The scapula is connected to the thoracic spine via the trapezius and the rhomboideus muscle so that during one-handed smartphone use, unilateral traction forces could act laterally on the thoracic spine. This assumption is supported by another study demonstrating a significantly increased muscle activity (measured via EMG) in the trapezius muscle during one-handed smartphone use [10]. The fact that a lower lateral deviation was observed under S2H supports this thesis since the tensile forces act equally from both sides on the spine.

In addition to the analysis of the spine, the gait parameters stride length, cadence, step width, and foot rotation were examined simultaneously. However, no gait changes were detected under the different conditions. It was noticeable that the subjects adjusted their gait due to the slow pace [25]. The reasons for this observation might be an insufficient challenge due to the selected speed of 3 km/h. Due to the treadmill situation, there were no obstacles during the walk, which is why the view and concentration could be completely focused on the smartphone. Accordingly, the subjects had sufficient resources despite the additional task to maintain a safe gait. However, smartphone-induced changes in gait were found in other studies at participant-selected speeds or while free walking without a treadmill [12,13].

Future studies should aim to explore the effects of smartphone use while walking in real-world environments or at higher speeds. The potentially associated increase in fall risk is of great concern, especially for older adults. In addition, similar studies should be conducted with women. It would also be interesting to evaluate the effects after prolonged smartphone use (e.g., several minutes) with multiple measurements of the spine on specific characteristics of the lumbar spine. In this way, a possibly occurring postural change caused by muscular fatigue can be presented.

### Strengths and Limitations

The major strength of the study is that it was possible to show changes in spinal posture during smartphone use that are expressed below the cervical spine. This study unveils, for the first time, alterations in the thoracic as well as lumbar spine contingent upon how the smartphone is held while standing or walking. These novel findings should be replicated in further studies.

We also need to acknowledge some limitations. The standardized speed of the treadmill of 3 km/h was rather slow and might not have been sufficiently challenging to induce gait changes in the selected sample. Further, the walking testing took place in a laboratory on a treadmill leading to the fact that no real-life environment was created and results regarding the gait as well as spine analyses are only in part transferable to smartphone use outside the laboratory. In addition, only young male adults participated in this study. Finally, the consideration of pressure distributions and center of pressure kinematics during smartphone use represents an interesting research perspective.

## 5. Conclusions

The influence of smartphone usage on the human organism is multifaceted. The findings acquired in this study provide new insights into the effects of smartphone use on the thoracic and lumbar spine. Specifically, an increased kyphosis angle in the thoracic spine and an increased VP-flexion were observed while reading a text on a smartphone held one- or two-handed, which are larger compared to normal values. Long-term smartphone usage in a position as observed in the present study might represent a risk factor for abnormal or even pathological posture changes (e.g., round back). In addition, resulting increased pressure and shear forces acting on the vertebral bodies, intervertebral discs, and muscles, might increase the risk of spinal pain. Moreover, an increased lateral deviation in the thoracic spine was detected during one-handed smartphone use. Regarding the lumbar lordosis angle, no significant changes were shown in the present study. Similar findings apply to the gait. The fact that in Germany, 75% of the population uses a smartphone, including more than 90% of 14–59-year-olds, shows that a large part of society is affected by this phenomenon. The societal impact and potentially incurred health care costs from treatments for cervical and thoracic complaints could be extensive. Accordingly, it is important to provide education at an early stage to recognize and prevent excessive smartphone use and addiction-like behavior. In addition, it seems important to recommend physical activity as a balance to the increasing screen-bound time to strengthen physical resources at an early stage.

## Figures and Tables

**Table 1:** Results of mean comparisons of kyphosis angle, lordosis angle, VP-flexion, and lateral deviation under standing and walking conditions for GN, S1H, and S2H.

					Repeated-Measures ANOVA	Post Hoc *p*-Value (Bonferroni Correction)		
Variables		GN (*n* = 20)	S1H (*n* = 20)	S2H (*n* = 20)		GN vs. S1H	GN vs. S2H	S1H vs. S2H
Kyphosis angle [°] ICT–ITL (max)	standing	49.1 ± 4.6 (41.1–58.0)	58.8 ± 5.8 (49.5–72.1)	61.6 ± 4.9 (53.9–70.5)	F(1.63, 32.68) = 147.01, *p* ≤ 0.001, η^2^ = 0.88; Power = 1.0	≤0.001	≤0.001	≤0.001
	walking	47.6 ± 4.6 (37.9–59.0)	56.5 ± 5.5 (45.7–67.0)	58.5 ± 5.6 (48.1–69.5)	F(2, 38) = 80.63, *p* ≤ 0.001, η^2^ = 0.81; Power = 1.0	≤0.001	≤0.001	≤0.001
Lordosis angle [°] ITL–ILS (max)	standing	35.5 ± 7.1 (21.4–49.8)	38.0 ± 7.6 (23.8–53.6)	37.6 ± 11.2 (23.3–75.5)	F(1.44, 28.79) = 0.96, *p* = 0.368, η^2^ = 0.46; Power = 1.0	0.150	1.000	1.000
	walking	31.1 ± 7.8 (14.9–43.9)	31.6 ± 7.7 (17.4–46.7)	32.4 ± 7.7 (20.3–46.9)	F(1.37, 25.96) = 1.97, *p* = 0.170, η^2^ = 0.09; Power = 0.84	0.871	0.429	0.615
VP-Flexion [°]	standing	17.6 ± 3.8 (8.9–25.2)	23.8 ± 6.9 (12.5–35.5)	22.4 ± 4.7 (14.1–30.6)	F(1.45, 28.95) = 17.30, *p* ≤ 0.001, η^2^ = 0.46; Power = 1.0	≤0.001	≤0.001	0.955
	walking	28.5 ± 7.6 (12.5–46.5)	35.9 ± 8.3 (15.6–48.8)	39.4 ± 8.3 (17.7–48.7)	F(2, 38) = 22.54, *p* ≤ 0.001, η^2^ = 0.54; Power = 1.0	0.003	≤0.001	0.043
Lateral Deviation [mm] VP–DM (RMS)	standing	4.6 ± 2.6 (1.1–11.7)	6.0 ± 4.0 (1.0–15.9)	3.5 ± 1.7 (1.0–7.1)	F(2, 40) = 5.87, *p* = 0.006, η^2^ = 0.23; Power = 1.0	0.349	0.198	0.009
	walking	5.4 ± 2.2 (1.6–10.4)	6.9 ± 3.6 (2.9–13.4)	5.6 ± 2.4 (2.3–11.5)	F(2, 38) = 3.3, *p* = 0.047, η^2^ = 0.15; Power = 0.98	0.334	1.000	0.018

Data presented as mean ± standard deviation (minimum–maximum). GN = gaze neutral; S1H = smartphone held one-handed; S2H = smartphone held two-handed; ° = degree; VP = vertebra prominens; DM = dimple middle; ICT = cervico-thoracic junction; ITL = thoraco-lumbar junction; ILS = lumbo-sacral junction; RMS = root mean square; η^2^ = partial eta square; Power = 1 − β. The *p*-values are calculated according to a repeated-measures ANOVA with Bonferroni correction.

**Table 2:** Results of gait analysis under the GN, S1H, and S2H viewing directions.

Variables	GN (*n* = 21)	S1H (*n* = 21)	S2H (*n* = 21)	Repeated-Measures ANOVA
Stride length (cm)	67.3 ± 4.4 (59.7–74.6)	67.7 ± 2.4 (63.4–73.0)	66.2 ± 3.4 (60.8–73.4)	F(1.49, 28.88) = 1.86, *p* = 0.180, η^2^ = 0.09; Power = 0.86
Cadence (steps/min)	88.6 ± 6.9 (76.2–101.7)	88.6 ± 7.2 (70.6–101.1)	87.3 ± 6.8 (71.6–100.8)	F(1.63, 32.60) = 0.45, *p* = 0.601, η^2^ = 0.02; Power = 0.25
Step width (cm)	9.7 ± 2.4 (4.5–14.1)	10.0 ± 2.1 (6.7–14.8)	9.9 ± 3.2 (2.4–15.6)	F(2, 40) = 0.16, *p* = 0.883, η^2^ = 0.006; Power = 0.15
Foot rotation right (°)	8.5 ± 5.3 (−5.5–18.3)	7.7 ± 4.0 (0.01–16.1)	7.6 ± 4.1 (0.8–14.5)	F(1.25, 25.04) = 0.42, *p* = 0.566, η^2^ = 0.02; Power = 0.25
Foot rotation left (°)	6.5 ± 6.0 (−6.3–17.8)	6.5 ± 6.4 (−7.8–17.1)	6.0 ± 6.2 (−5.1–16.2)	F(1.17, 23.40) = 0.77, *p* = 0.822, η^2^ = 0.004; Power = 0.05

Data presented as mean ± standard deviation (minimum–maximum). GN = gaze neutral; S1H = smartphone held one-handed; S2H = smartphone held two-handed; ° = degree; η^2^ = partial eta square; Power = 1 − β. The *p*-values are calculated according to a repeated-measures ANOVA with Bonferroni correction.

## Data Availability

The data presented in this study are available on request from the corresponding author.
